# Market introduction of plant varieties and products with gene-edited traits

**DOI:** 10.1080/21645698.2026.2684804

**Published:** 2026-06-21

**Authors:** Joanna M. Lukasiewicz, Marinus J. M. Smulders

**Affiliations:** Plant Sciences Group, Wageningen University & Research, Wageningen, The Netherlands

**Keywords:** Consumer acceptance, gene editing, GM crops, GMO regulation, plant varieties

## Abstract

Gene editing technologies can be used to develop new traits in plants by modifying DNA at specific locations. In many countries, plants made using these techniques, collectively called New Genomic Techniques (NGTs), are being regulated more leniently than transgenic genetically modified organisms (GMOs), opening possibilities for releasing gene-edited plant varieties onto the market. In other countries, including the European Union (EU), proposals for an adapted regulation are in discussion. We describe how the different types of modifications made with NGTs are regulated across the world. We provide an updated overview of gene-edited plant products that have been marketed worldwide, approved, or are currently in field trials. Consumer perception may be a challenge for plant products made using NGTs, but otherwise the commercialization of these new varieties faces the same challenges as new conventional products, including farmer and processor needs, market competition, and consumer preferences.

## Introduction

**Figure 1.**
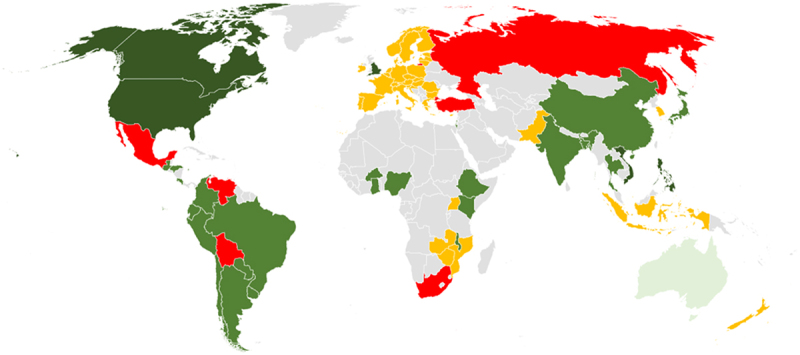
Regulation of plant products developed using NGTs across the world. Categories are made based on the SDN groups: SDN-1 defines point mutations made using no template, SDN-2 are point mutations using a templates, and SDN-3 contains cisgenic products with larger insertions. Dark green countries (canada, England, the Philippines, the United States of America, Vietnam) regulate these products as conventional products, no separate assessment of new gene-edited products is needed. Transgenic SDN-3 remains under GMO regulations. In the case of Vietnam, developers determine themselves if they fall under GE or classical GMO regulation. In Canada, products are deregulated if no novel traits are present. In countries marked Green (argentina, Bangladesh, Brazil, Burkina Faso, Chile, China, Colombia, Costa Rica, Ecuador, Ethiopia, Ghana, Guatemala, Honduras, India, Israel, Japan, Kenya, Malawi, Nigeria, Paraguay, Peru, Singapore, Thailand, Uruguay) products are assessed on a case-by-case basis by national competent authorities. SDN-1, SDN-2, and cisgenic SDN-3 are predominately approved, while transgenic SDN-3 products remain under GMO regulations. Light green countries: in Australia, only SDN-1 is regulated as conventional products; SDN-2 and SDN-3 remain under GMO regulations. Yellow countries (The EU, Indonesia, Mozambique, new-Zealand, Norway, Pakistan, South-Korea, Switzerland, Uganda, Zambia, Zimbabwe): discussions are underway or proposals have been made about the use of NGTs in plant breeding. Red countries (bolivia, Mexico, Russia, South-Africa, Türkiye, Venezuela): NGTs in plant breeding are strictly regulated or regulated as GMOs (if GMO regulation is in place); or gene editing techniques are only used for research purposes (notably Russia). This Figure was made in using Microsoft excel, based on information gathered by the first author. The internet page http://cropgeregulations.com contains a comparable overview with the intention of keeping it up to date.

Crop breeding is based on crossing and selecting genotypes with desirable traits. Mutations generate the genetic variation with which breeders work. These mutations may have arisen naturally (the standing genetic variation in crops and their wild relatives) or can be generated through random mutagenesis techniques by applying, e.g., radiation or mutagenic chemicals.^1^ Selection for advantageous mutations in the plants generated using these random mutagenesis methods is inefficient, especially for traits controlled by multiple genes or in polyploid crops and/or a crops with a long generation cycle.^2^ Genetic modification or gene editing has enabled the developed of crops with targeted mutations or inserted genes. The insertion of genes into plant cells was made possible by the discovery of *Agrobacterium tumefaciens* as a carrier of genetic material.^3^ Genetically Modified Organisms (GMOs) can contain inserted genes from a non-crossable species, so-called transgenes, or genes from a crossable species, cisgenes.^4^ The insertion of genes using *Agrobacterium tumefaciens* occurs in a random place in the genome. With the advent of gene editing techniques or New Genomic Techniques (NGTs), developed in the last twenty-five years, targeted mutagenesis became possible.^5^ The majority of these techniques are based on nucleases that cut DNA (Box 1) and small mutations occasionally produced by imperfections of the cell’s DNA repair mechanisms can be selected for among the regenerants.^6^ Several countries worldwide have introduced a more lenient regulation of NGTs compared to regulation of transgenic GMOs (Figure 1 & Table 1), in which gene-edited plants that contain no transgenic material are mostly regulated similarly to conventional products. Countries that have approved gene-edited plants apply a “case-by-case assessment,” in which the national competent authority determines how a modified plant or product is regulated (Box 2).

**Box 1. New Genomic Techniques in plant breeding**

New Genomic Techniques (NGTs) are gene editing tools that were discovered or developed in the last 20 years. Apart from Oligo-Directed Mutagenesis (ODM), the techniques involve nucleases that can cut DNA at specified locations, such as Meganucleases, ZINC Finger Nucleases (ZFNs), Transcription Activator-Like (TAL) Effector Nucleases (TALENs) and, the mostly widely used, Clustered Regularly Interspaced Short Palindromic Repeats (CRISPR) combined with a CRISPR-associated protein (Cas).

CRISPR-Cas is easy to use and efficient ^7^ and it has rapidly revolutionized the field of gene editing. It consists of a CRISPR-associated (Cas) enzyme and a guide RNA (gRNA) sequence that together form a nuclease complex. The Cas enzyme scans the DNA for Protospacer Adjacent Motifs (PAMs) in the genome. When a PAM is present and the upstream region is complementary to the gRNA sequence, the Cas enzyme cuts the DNA. In further developments, Cas enzymes have been modified to cut only a single strand, and/or to modify specific nucleotides in the process. For base editing, a Cas nickase is fused to a cytidine or adenosine deaminase that can substitute nucleotides leading to changed or truncated proteins.^8,9^ Prime editing is able to rewrite short DNA sequences by fusing a transcriptase to the Cas nickase.^10^

Meganucleases, ZFNs and TALENs also cut DNA, but they employ larger protein structures that are custom designed for each target, leading to lower flexibility in using the tool.^11^ ODM is not based on the use of a nuclease, instead, it is a single-stranded DNA string that may induce small nucleotide mutations when coupled to the sense strand during DNA repair.^12,13^ It is not widely used as it is relatively inefficient.

Targeted mutagenesis (the use of NGTs to make mutations at predefined positions) may be used in combination with cisgenesis to insert species-specific genes in predefined locations in the genome. Cisgenesis is more easily accepted by society compared to transgenesis since the same result can be obtained by conventional breeding methods.^14^ Cisgenesis using targeted or random insertion is faster and more efficient compared to conventional breeding methods, especially in vegetatively propagated crops such as potato and banana.^15^

Targeted mutagenesis or targeted cisgenesis result in plants that can be considered equivalent to plants obtained by conventional breeding methods.^16^ The European Food Safety Authority (EFSA) has therefore concluded that no additional risks are expected from these products. In the case of cisgenesis an additional provision is required: the insert should not disrupt an endogenous gene.^16^

**Box 2. Gene editing terminology: Side-Directed Nuclease (SDN).**

Genetic modification is the commonly used term when referring to GMO plants and products developed since 1982, while the term NGTs (or New Plant Breeding Techniques (NPBTs)) encompasses techniques that have been developed after 2001. The term NGTs is most often used when referring to gene editing tools in the EU. Worldwide, the term Site-Directed Nuclease (SDN) is often applied when talking about regulation of gene-edited plants products. SDN is categorized into three groups, SDN-1, SDN-2, and SDN-3, based on the mutation introduced and on the use of an exogenous DNA template.^51–53^ SDN-1 includes products with small modifications arising from mistakes in the plant repair mechanisms after targeted mutagenesis using nucleases such as CRISPR-Cas, Meganucleases, TALENs, or ZFNs. Apart from the nuclease and gRNA for CRISPR-Cas, no exogenous DNA template is used. An example of a SDN-1 product is the GABA-tomato in Japan (Table 2). The modifications in SDN-2 are of the same order as in SDN-1, but in this case an exogenous DNA template is used to introduce the desired mutation (consisting of one or a few nucleotides). The introduction of template DNA enables the introduction of specific mutations at a desired location. ODM falls under SDN-2 since exogenous DNA is introduced and the size of the mutations is limited. Larger genetic insertions, either transgenic or cisgenic, are grouped under SDN-3.

In most countries that have specific regulations for gene-edited plant products in place, approval is determined on a “case-by-case” basis which means that the applicant supplies information to the national competent authority about the modifications made and the authority determines if it exempted from regulation. Several countries in Latin-America follow this “case-by-case” assessment including Argentina, Brazil, and Chile (Fig. 1 &amp ; Table 1).^87^ Overall, in countries that have a “case-by- case” assessment, SDN-1, SDN-2, and cisgenic SDN-3 are not considered as GMO and only registration of the gene-edited products is required. There are some exceptions. In Vietnam, the applicant self-determines if the plant or products falls under GE or GMO regulation.^39^ Australia only allows SDN-1, single point mutations, while SDN-2 and SDN-3 remain under GMO regulations.^32^ In Japan SDN-1 is exempted, while SDN-2 and SDN-3 are regulated on a “case-by case” basis.^40^ Japan is one of the countries that follows the Cartagena Protocol on Biosafety which states that living modified organisms (LMOs) are not regulated when RNA and recombinant proteins have been used.^40^ Costa Rica also regulates an organism as an LMO when it contains a novel combination of genetic material.^88^ In the USA, only transgenic plants and products are considered GMO and need to be labeled as “bioengineered” since 1 January 2022. Gene-edited plants and products that do not contain transgenic material and do not pose plant pest risks are approved for the market ^28^ Notifications are on a voluntary basis. In Canada, risk assessments are based on trait novelty determined by the applicant; novel foods need to pass a safety assessment.^89^ It is interesting to note that in the list of products with Novel Traits, herbicide-tolerance traits are often made using conventional breeding technologies.^27^

At the moment, only Japan and the USA have marketed gene-edited products which are directly available to consumers. These concern the GABA-tomato and the non-browning lettuce, both of which are sold as seed directly to consumers. A number of products have been approved in Canada.^27^ England has adopted the Precision Breeding Bill allowing gene-edited plants and animals to be marketed, which has entered into force in November 2025.^44^ A few gene-edited plant products have been accepted for the market and others can be expected in the near future (Table 2).

**Table 1.** Regulation of gene-edited plants and products in various countries. This information and the status is also shown in Figure 1.

Continent	Countries	Regulatory system	References
Africa	Burkina Faso, Ethiopia, Ghana, Kenya, Malawi, Nigeria	Evaluation by competent national authority needed before approval (“case-by-case assessment”). Only a novel combination of genetic material (including transgenesis) is considered a GMO (following the Cartagena Protocol on Biosafety ^17^)	18 19 20 21,22
Africa	Mozambique, Uganda, Zambia, Zimbabwe	Proposal or discussions ongoing on the regulation of gene editing (Mozambique, Zambia and Zimbabwe) or gene editing is at the moment only used for research purposes (Uganda)	23 24 25 26
Africa	South-Africa	Gene editing regulated under GMO guidelines	25
North America	Canada	Regulation based on novelty of the trait	27
North America	The United States of America	Plants and products that contain no transgenes and pose no plant pest risk are regulated as conventional plants and products	28
South America	Bolivia, Mexico, Venezuela	Gene editing regulated under GMO guidelines	29
South America	Argentina, Brazil, Chile, Colombia, Costa Rica, Ecuador, Guatemala, Honduras, Paraguay, Peru, Uruguay	Evaluation by competent national authority needed before approval (“case-by-case assessment”). In some cases, only a novel combination of genetic material (including transgenesis) is considered a GMO (following the Cartagena Protocol on Biosafety ^17^)	30 31
Oceania	Australia	SDN-1 is exempted. SDN-2 and SDN-3 are considered GMO	32
Oceania	New-Zealand	Proposal on allowing commercialization of gene-edited plants. Originally planned for end of 2025	33
Asia	Bangladesh, China, India, Singapore, Thailand	Evaluation by competent national authority needed before approval (“case-by-case assessment”). Gene-edited plants and products not regulated as GMO when no foreign DNA is present	34 35 36 37
Asia	The Philippines	Non-GMO if they do not contain a novel combination of genetic material in the final product (following the Cartagena Protocol ^17^). The developer has to request a technical Consultation for Evaluation and Determination (TCED)	38
Asia	Vietnam	Self-determination process: Notification to the national authority needs to be submitted by the applicant	39
Asia	Japan	SDN-1 exempted; “case-by-case assessment” for SDN-2 and (cisgenic) SDN-3. Only novel combination of genetic material is considered a GMO (following the Cartagena Protocol on Biosafety ^17^)	40
Asia	Indonesia, Pakistan, South-Korea	Proposal or discussions on the regulation of gene editing. Gene-edited products need to be labeled in Indonesia	41 42 43
Europe	England	Precision Breeding Act: Plants and Animal (Precision Bred Organisms) are not considered GMO	44
Europe	European Union	Proposal pending. No “case-by-case assessment”	45
Europe	Norway	Regulated as GMO at the moment, but this might change in the near future	46
Europe	Switzerland	Under a new proposal, gene-edited plants will be equivalent to those developed using conventional genetic engineering, as opposed to GMOs. Gene-edited products will need to be labeled.	47
Eurasia	Israel	Gene-edited plants and products are not regulated as GMO when no foreign DNA is present	48
Eurasia	Russia, Türkiye	Research is being conducted in these countries, but gene editing remains regulated under GMO guidelines	49 50

**Table 2.** Characteristics of the gene-edited crops that are currently on the market or have been approved for commercialization.

Crop	Trait	Technology*	Company	Country (Year)	Reference
Alfalfa	Modified lignin polymer composition	TALENs (SDN-1)	Calyxt Inc. (USA)	Canada (2022). Not intended for food use, but for forage; earliest market entry 2023.	27
Banana	Non-browning and disease resistance	CRISPR-Cas knockout of genes regulating polyphenol oxidase (SDN-1)	Tropic Biosciences (UK)	Approved for cultivation and import in the Philippines (2024), Japan, and Brazil (2026).	54 55 56
Barley	Higher forage potential	Editing of genes leading to higher lipid levels (SDN-1)	Rothamsted Research (UK)	Approved for marketing (2026) as a feed crop.	57
Blackberries	Improved quality (seedless and/or thornless)	CRISPR-Cas editing of transcription factors (SDN-1)	Pairwise (USA)	Earliest market entry in Canada in 2028.	27
Camelina	Increased oil content	CRISPR-Cas modification of three target genes (SDN-1)	Yield10 Bioscience Inc. (USA). Filed for bankruptcy in 2024.^58^	USA (2017) Argentina (2022) Canada (2024) Not commercialized.	59 60
Canola	Herbicide tolerance	ODM** (SDN-2)	Cibus Inc. (USA)	USA (2013) & Canada (2014). Field trials conducted in 2015 (no status update).	61 62
Canola	Disease resistance (*Sclerotinia*)	CRISPR-Cas (SDN-1)	Cibus Inc. (USA)	Planned in Canada (not approved yet for consumption).	63 64
Lettuce	Non-browning	CRISPR-Cas (SDN-1)	GreenVenus^tm^ (USA)	Sold as seed directly to consumers in the USA (2020). The company is also working on other products.	65
Maize (“Waxy Maize/Waxy Corn”)	Higher levels of amylopectin	Editing of Wx1 using CRISPR-Cas (SDN-1)	Corteva Agriscience (subsidiary: Pioneer Hi-Bred) (Canada/USA)	Japan (2023) and several other countries (incl. Canada and the USA). Approved, but not commercialized yet. Field trials in the USA.	66 67 27
Maize/Corn	Fungal resistance	Cisgenesis (SDN-3)	Pioneer Hi-Bred Canada company	Approved in Canada (commercialization planned for 2028).	27
Mushrooms	Non-browning	CRISPR-Cas	Penn State University (USA)	Approved (2016), but not commercialized.	68
Mustard greens (“Conscious Greens”)**	Less pungent mustard greens	CRISPR-Cas (SDN-1) deletions and insertions in a gene involved in the breakdown of glucosinolates	Pairwise (USA)	Marketed in the USA (2023), and approved in Canada (2023). Discontinued in 2024 after less than a year.	69 27 70
Pennycress	Altered oil composition/higher yield	CRISPR-Cas	Covercress Inc./Illinois State University (USA)	Approved in the USA (2024). Not commercialized yet.	71
Potato	Higher tuber yield	CRISPR-Cas editing of *Gn2* gene (SDN-1)	J.R. Simplot (USA)	Approved in Canada, USA, and Japan (2024). Not commercialized yet.	27 72
Potato	Reduced PPOs (reduced browning)	CRISPR-Cas (SDN-1) deletions	J.R. Simplot (USA)	Approved in Canada (2024). Not commercialized yet; earliest market entry 2025.	27
Soybean (“Calyno^tm^” and “HOLL”)	High oleic/low acidic soybean oil***	TALENs	Calyxt Inc.**** (USA)	Calyno^tm^ was approved in USA and Canada (2019). Unclear if it is still on the market.	73 74 75
Soybean	Increased yield through changes in architecture	CRISPR-Cas editing of several genes (SDN-1)	Inari Agriculture Inc. (USA/Belgium)	Earliest market entry in Canada in 2026.	27
Soybean	Herbicide tolerance	CRISPR-Cas	Inari Agriculture Inc. (USA/Belgium)	Earliest market entry in Canada in 2027.	27
Strawberry	Longer harvest time	CRISPR-Cas deletions (SDN-1)	J.R. Simplot/Plant Sciences Inc. (USA)	Approved in Canada (2025). Not commercialized yet.	27
Tomato (“Sicilian Rouge”)	Higher GABA levels	CRISPR-Cas knockout of *GAD* genes (SDN-1)	Sanatech Seed (Japan)	Commercialized in Japan (2021). Sold as seed directly to consumers. Approved in the Philippines,^76^ and Singapore,^77^ and submitted for approval in Canada.^78^	79 80
Tomato	Longer shelf-life	CRISPR-Cas knockout of pectate lyase genes to reduce cell wall breakdown (SDN-1)	Lark Seeds International (USA)	Earliest market entry in Cananda in 2028.	27
Wheat	Powdery mildew resistance	CRISPR-Cas editing of *MLO* gene (SDN-1)	Chinese Academy of Sciences (China)	Approved in China (2024).	81 82 83
Wheat	High fiber	CRISPR-Cas (SDN-1)	Neocrops technologies (Chile/Argentina)	Approved in Argentina (2025).	84

* SDN categories inferred by the authors to the best of their knowledge.

** The origin of this herbicide-resistant canola was changed to arising from a somaclonal mutation through tissue culture regeneration ^27^.

*** China has also approved a GE high-oleic soybean in 2023 ^85^.

*** Many other approved gene-edited products (such non-browning potato, high-fiber wheat and powdery mildew resistant wheat ^86^ from Calyxt Inc. were discontinued after the company was merged to form Cibus Inc. in 2023.

Plants with insertion of transgenic material or, in some countries, where external transgenic templates have been used to generate a mutation, often remain under stricter GMO regulations. Some countries however, notably Canada, England, Vietnam, the USA, and the Philippines, have developed guidelines that specify when a gene-edited plant is deregulated; while a notification might be required, a separate assessment is not needed. In some cases, registration of the NGT plant or product in a public database is required, although in some cases this is on a voluntary basis (e.g., in the USA and Canada). However, in a large number of countries where transgene-free plant products have been deregulated no registration is required (e.g. in China and countries in Latin America). This means that it is difficult to identify products that have been gene-edited in these countries since they fall under conventional products and do not need a separate categorization.

Some countries are in the process of evaluating proposals to modify the regulations (Table 1). In the European Union, a proposal has been made by the European Commission (EC) to deregulate plants made using NGTs (Box 3).

**Box 3. The European Commission’s proposal for an adapted regulation of NGTs.**

After the ruling of the Court of Justice of the European Union (CJEU) in 2018 that plants made by NGTs fall under the GMO Directive for Deliberate Release (2001/18/EC), the European Commission (EC) determined (after a requested study by the European Council), that the GMO Directive is not “fit for purpose” for plants made using NGTs. Therefore, the EC proposed an adapted regulation of NGTs in July 2023.^45^ Certain breeding techniques, such as random mutagenesis, are already exempted from the GMO Directive. The proposal considers two new categories. NGT-1 plants are considered to be equivalent to plants derived from conventional breeding methods and random mutagenesis methods, and will therefore be exempted from the GMO Directive. NGT-1 plants and products will not be labeled, apart from seed and reproductive material. Certain traits, herbicide-tolerance and the production of a known insecticidal substance, will be excluded from the NGT-1 category. NGT-2 plants and products contain a higher number of modifications, or modifications that do not fall under NGT-1. These plants and products will have an adapted risk assessment.^45^ Neither category will be allowed in organic farming. The European Parliament (EP) voted in favor of the proposal with several amendments; the Council, EC, and EP have made a compromise and produced a consolidated proposal during Trilogue discussions. At time of writing, a provisional agreement has been made public by the Council.^90^

It is important to note that the NGT categories envisioned by the EC are different from the SDN terminology which is used in several countries outside of the EU. This means that there may be a discrepancy in import and export of these plant products. Harmonization between regulation and terminology may be desired in the future.

In the proposal, a distinction is made between plants that can be considered equivalent to plants made using conventional breeding techniques (classified as NGT-1), and plants with a larger number of mutations (classified as NGT-2).^45^ Recently, an agreement has been reached between the Council and the European Parliament, thus getting closer to finalizing a new regulation for NGTs.

## “Classical” GMO Traits

Most GM crops have traits that are of benefit to growers, the most popular GM traits being herbicide tolerance and insect resistance through the *Bt* gene,^91^ and these occupy the great majority of the 206.3 million hectares planted in 27 countries in 2023.^92^ Herbicide-tolerant maize and soybean provide the farmer with more flexibility in weed management, while insect resistance in maize and cotton reduces damage and allows a lower use of pesticides. Both reduce cultivation risks and thus fulfil a need for the farmer. Other GM crops with traits for farmers include BT brinjal (eggplant) cultivated in Bangladesh,^93^ virus resistant papaya cultivated in the USA and China,^94^ and drought-tolerant soybean and sugar cane.^95^

The first GM crops were actually addressed to consumers. The Favr Savr tomato, developed by Calgene, has a modified trait (longer shelf-life) that was interesting to distributors as well as consumers. it has lower polygalacturonase enzyme levels, leading to reduced pectin degradation in the fruit cell walls, delayed softening and extending shelf-life.^96^ Initially, the Flavr Savr tomato sold well on the fresh market in California from 1994 onwards and as canned paste in the UK with a GM label from 1996 to 1999, successfully competing with a 20% lower price due to lower processing costs (the paste also had a higher viscosity). However, despite positive consumer interest, the fresh market for the Flavr Savr tomato in California faltered due to high production and distribution costs,^97^ as Calgene was inexperienced in tomato growing, handling, and transport. The Flavr Savr tomato disappeared from the shelves in 1997.^98^ From 1998 onwards also sales of canned tomatoes dropped due to concerns about GM as a result of negative media coverage. Calgene was subsequently bought by Monsanto.

Similarly, other GM products were marketed with traits tailored toward consumers. These include traits that lead to non-browning, a novel color (blue/purple carnation) or have an increased nutrient composition (pink pineapple, “Golden Rice”). For example, the Arctic Apple® is an apple variety that does not turn brown when cut. These apples are sold as sliced apple pieces in, for example, lunch packages. It has been developed in Canada by Okanagan Fruits Inc. and was approved in the US in 2015 as a GM product.^99^ Okanagan Fruits Inc. also plans to develop a non-browning apple using NGTs. This product would not contain a transgenic insertion for gene silencing and thus not classify as transgene.

In 2023, a new GM product targeted consumers in the USA. This was a purple tomato containing an anthocyanin gene from snapdragon (an edible flower) developed by Norfolk Plant Sciences.^100^ Anthocyanins are antioxidants that have anti-cancer and anti-inflammatory properties.^101^ Similarly to the NGT products GABA tomato and non-browning lettuce, the seeds are directly sold to home gardeners as customers.

## NGT Products on the Market or Approved for Commercialization

As of 2025, to our knowledge, four NGT plant products are on the market (high-oleic, soybean oil, non-browning lettuce, “Conscious Greens,” and the GABA tomato) (Table 2). The tomato with increased GABA levels has been developed by the Japanese company Sanatech Seed.^80^ These gene-edited “Sicilian Rouge” tomatoes are sold as seed to small household growers as a new product with beneficial health properties. By selling the gene-edited plants directly to consumers, Sanatech Seed bypasses distributors and retailers, parties that might have been unwilling in selling the gene-edited products.

The company Pairwise, in collaboration with Corteva Agriscience, works on developing varieties of smaller crops with gene-edited traits. Their first product, “Conscious Greens,” a less pungent mustard green, that has been approved for the market in 2023, but it has been removed from production despite positive consumer responses. This is due to a lack of an established position in the salad market, according to Pairwise.^69^ Other products from Pairwise are in the pipeline such as seedless berries and cherries. The company GreenVenus^tm^ produced a gene-edited lettuce to introduce the non-browning trait leading to a longer shelf-life. Similarly to the tomato from Sanatech Seed, the seeds of this lettuce are sold directly to consumers.

Several other gene-edited plants and products have been approved for the market (including in countries such as Canada, the USA, Japan, and the Philippines), but have not been marketed, such as the non-browning mushrooms developed by Penn State and the gene-edited camelina by Yield10 Bioscience Inc. (this last company went bankrupt in 2024) (Table 2). There are also products being developed in China and several South American countries (including Brazil and Argentina), but finding relevant information on these products was not straightforward. In our experience, it is challenging to determine if approved products are eventually commercialized. If not, it is even more difficult to determine why, since the reasons to not follow-up with commercialization are diverse and are generally not made public by companies.

The high-linoleic soybean oil (Calyno^tm^) of Calyxt Inc. is one of the first products that have been commercialized (in 2019, but currently not available). Calyxt Inc. also developed high oleic acid canola and high oleic/low linolic soybean (“HOLL”). Both these products have been approved, but not commercialized. Calyxt Inc. had other approved products (high-fiber wheat, powdery-mildew resistant wheat, improved quality alfalfa, cold storage potatoes and reduced browning potatoes),^86^ which have not been commercialized after the merger into Cibus Inc. Gene-edited waxy maize,^66^ with increased amylopectin levels has been cleared for commercialization in the USA, Canada, Japan, and several other countries.^67^ While these products have been cleared, they are not commercially available (yet).

Products that have been recently approved (since last year) may be commercialized in the coming years (Table 2). A non-browning banana developed by Tropic Biosciences in the UK has been approved for cultivation in the Philippines. Tropic Biosciences also works on disease resistance in banana and on gene editing other crops such as coffee and rice. Various disease-resistant products are in the pipeline such as rice (from Cibus Inc.), maize, and potato (from J.R. Simplot). It may also be expected that several future gene-edited plants will be modified for herbicide tolerance, as it is a trait much desired by farmers as it simplifies weed management and reduces cultivation risks. The Canadian list of non-novel foods includes several plants produced with herbicide tolerance developed using conventional breeding and classical mutagenesis.^27^

Even though several countries have approved gene editing technologies to make new crops, only a few products have already been commercialized in the past years. There are several new gene-edited products approved, and it remains to be seen if their market introduction will be successful. Looking at the gene-edited traits that are currently being marketed, the majority is tailored toward consumers and is sold directly as seed (the GABA tomato and the non-browning lettuce), this can be compared to smaller GM products such as the purple GM tomato which is also sold as seed.^100^ Major GM crops have traits benefitting produces such as herbicide tolerance and insect resistance. These traits are not yet represented in gene-edited products that are in the pipeline, but it may be expected that these traits will also be incorporated in gene-edited products. So far, the majority of these gene-edited products are developed by smaller biotech companies (GreenVenus^tm^, Cibus Inc., Pairwise, Sanatech Seed, Tropic Biosciences), rather than by larger seed breeding companies (e.g., Corteva Agriscience, Bayer Crop Science, BASF, J.R. Simplot). This might explain why early gene-edited products have a smaller market share.

To summarize, the first gene-edited plants and products that have been marketed are developed by smaller biotech companies, and not the larger seed breeding companies, although J.R. Simplot has filed for approval of several gene-edited products. These companies have a smaller scope compared to larger companies which have a larger leverage toward logistical and retailers. The lack of farmer-engagement for these early products might also explain the focus toward consumer-orientated traits such as longer shelf-life (non-browning) and a higher nutritional value. This may change as more countries adopt more lenient regulations toward gene editing technologies, leading to products developed by larger companies and more farmer-orientated traits such as higher yield and disease resistance.

Furthermore, it is difficult to determine the reason(s) why products in the pipeline are never marketed. There may be several reasons: either no protection of the variety or trait was granted, no market for cultivation and commercialization was found, no supply-chain was established, or investment were re-directed toward other causes. Once commercialized, a product can also fail due to low consumer demand.

In the case of the “Flavr-Savr” tomato, commercialization failed due to logistical problems, while the gene-edited “Conscious Greens” of Pairwise was retracted to focus on other products. In other cases, approved products were not commercialized due to bankruptcy of the company (e.g. Yield10) or mergers (e.g. Calyxt Inc.). It is interesting to see how these factors will influence commercialization and market acceptance of products developed by larger, established seed breeding companies.

## Field Trials of Gene-Edited Crops in Europe

No NGT plants or products are yet available on the European market, but several research trials of gene-edited crops have started or are in the pipeline (Table 3). Trials of modified wheat with lower asparagine levels have been conducted in England,^114,116^ which adopted the Precision Breeding Bill in 2020, allowing gene editing technology in farm animals and crops.^44^ A couple of trials with gene-edited plants are also conducted or planned in the EU. A fungus-resistant rice variety and grapevine were tested in Italy,^105^ but both field trials were destroyed.^117,118^ More applications for field trials with gene-edited plants have been filed in, amongst other countries, the UK, France, and the Netherlands (personal communication). In the Netherlands, a GMO field trial containing gene-edited potato was started in 2026. Field trials with Phytophthora-resistant potatoes are also planned in Norway and Switzerland. Field trials with GMOs can be consulted in the GMO register of the EC.^119^

**Table 3.** Selection of field trials of gene-edited crops (either underway or applied for). A selection was made to exemplify field trials of major crops in various countries; this list is not comprehensive. To the best knowledge of the authors, all examples fall under SDN-1 unless otherwise specified.

Crop	Trait	Technology	Company/Institute	Reference
Banana	Increased vitamin A	CRISPR base editing of the LCYɛ gene leads to enhanced beta-carotene content	National Agri-Food Biotechnology Institute (India).	102
Camelina	Increased seed size, yield, and oil content	CRISPR-Cas editing of genes controlling cell division	Rothamsted Research (UK).	103
Conifer	Improved timber quality	CRISPR-Cas editing of two separate genes	Scion group (Bioeconomy Science Institute) (New Zealand).	104
Grapes	Disease resistance (Downy Mildew)	CRISPR-Cas editing of two *DMR6* genes	EdiVite, Italy. Field trial destroyed.	105
Grapes	Non-browning, longer shelf-life, better tasting	CRISPR-Cas knockout	GreenVenus^tm^ (USA). Field trials.	65
Maize	Higher digestibility and climate adaptability	CRISPR-Cas editing of lignin genes; editing of DNA (folding) components for higher drought tolerance	VIB and ILVO (Belgium). Field trials were announced in 2022, but no follow-up news has been released.	106
Poplar	Lower lignin levels to simplify paper production	CRISPR-Cas editing of lignin genes	VIB and ILVO. Field trials planned in Belgium. No updates.	107
Potato	Disease resistance	CRISPR-Cas editing of various *Susceptibility* genes	SLU (Swedish University of Agricultural Sciences) in the GeneBEcon project. Field trials conducted in Sweden.	108
Potato	Disease resistance against *Phytophthora*	Cisgenic potatoes and gene knockouts using CRISPR-Cas	Wageningen University and Research (the Netherlands). Field trials started in 2026.	109
Potato	Disease resistance against *Phytophthora*	Cisgenic potatoes	Agroscope (Switzerland).	110
Potato	Disease resistance against *Phytophthora*	Gene-edited potatoes	The Sainsbury Laboratory (England) and NIBIO Norwegian Institute of Bioeconomy (Norway). Field trials planned for 2027.	(J.M.L., personal communication)
Rice	Lower susceptibility to rice blast fungus	CRISPR-Cas editing of Pi21 leading to the risotto variety “RIS8imo”	University of Milan, Italy. Field trials destroyed.	105
Rice	Drought and salt resistance (“DST1”) and higher yield (“DRR Dhan 100”)	CRISPR-Cas editing of the *DTS* gene (drought and salt resistance) leading to broader leaves and a lower density of stomata, and editing of a cytokinin gene (*OsCKX2*) for higher yield	Indian Agricultural Research Institute (India). Field trials underway. Two varieties approved.	111 112 113
Sorghum	Striga disease resistance	CRISPR-Cas knockout of locus related to Striga attachment	Kenyatta University (Kenya/ PennState/Corteva).	25
Wheat	Lower acrylamide levels	CRISPR-Cas *TaSN2* knockout (SDN-1)	Rothamsted Research (UK). Field trials previously conducted in England. Heated flour of gene-edited wheat had 45% less acrylamide.	114 115

## Future Trends

In countries (e.g., the USA, Canada, and Japan) where regulations have made commercialization of gene-edited plants and products, the number on the market is still limited. However, according to products listed on the novel food list in Canada,^27^ new products can be expected, including those developed by larger companies. Moreover, many other potential gene-edited products are in the research phase in other parts of the world, including crops that are interesting for non-Western markets such as disease-resistant cacao, sorghum, cassava and cowpea.^120^ Gene editing will also speed up the development (domestication) of new crops (e.g., ^121^). It remains to be seen, however, how successful these products will be for consumers, retailers, processors, and farmers.

## Market Factors That May Determine Success

NGTs often hold the promise that they lead to more sustainable crops, i.e., crops that are resilient to pests and diseases or drought-tolerant. While some traits are relatively easy to modify, such as fruit color by knocking out genes, or disease resistance by inserting cisgenic resistance genes or knocking out susceptibility genes, other traits require more research and are more difficult to generate as these traits are controlled by various genes or gene networks. Examples are salt tolerance and drought resistance.^95,122^

Modified traits generally are of interest to some parties in the food chain, but not to all. For example, a higher yield, disease resistance, or herbicide tolerance have direct advantages to farmers, but the end-consumer does not see the benefits. Many of the early GM crops have modifications that are beneficial to growers, such as herbicide tolerance.^123^ Other traits might be more applicable to distributors, such an extension of shelf-life or a change in ripening time, or to processors, such as lower levels of acrylamide in potatoes (thus eliminating a costly processing step). Plant traits that are interesting to the consumer can be based on nutrient level (such as the GABA tomato) or on visual characteristics (such as the blue/purple GM carnation), while these traits do not give an immediate benefit to the other parties in the chain. These new products need to be picked up by farmers to start producing them and a production chain needs to be in place that separates it from regular products. However, setting up a whole new production chain for a new NGT product might be expensive, risky or complicated, while the added value may not be distributed equally along the chain. With this in mind, it is noteworthy that in Japan the GABA tomato is marketed as seeds to be grown in home gardens.^124^ Thereby the breeder directly sells the seeds through channels he knows or already uses, as many plant breeding companies originated as producers and sellers of seeds and still have a large seed-producing facility and a commercial department selling the seeds. Some companies sell directly to consumers or have a brand of seed packaged for consumers and sold in, e.g., garden centers. In this way, the breeder can market directly to the group that may benefit from the product, thus circumventing having to set up a production chain in which such products should be traded separately and labeled to be visible in shops.

A successful introduction of a new variety not only depends on consumer acceptance, but also on other factors that conventional plant varieties and products also face, such as farmer and processor needs, competition with other varieties of the same crop and with similar products, market dynamics, and distribution limitations. Overall, most newly introduced varieties do not become a success.

There are several studies on consumer acceptance of gene-edited products as opposed to GMOs. The modified trait, the modification itself (transgenesis vs cisgenesis and smaller mutations), and the modified organism (plants vs animals) all influence consumer acceptance. Traits related to sustainability, e.g., disease-resistance, are generally seen more favorably by consumers. Younger respondents are more positive toward gene editing technologies.^125^ In a Finnish consumer and farmer study, the sense of what naturalness entailed was important. Consumers were more open to gene editing when the result resembled conventional or traditional methods. Farmers on the other hand, were more focused on economic implications from NGTs.^126^ The regulatory landscape also plays a role, since consumers in the United States had a more favorable perception toward gene-edited plants than consumers in Switzerland.^127^ More market exposure can have a positive effect on consumer perceptions of gene-edited products.

Apart from developing varieties as completely new products, and then hoping to gain a market share, breeders may also focus on improving existing varieties. This may be especially attractive for crops that are vegetatively propagated, which is the way to multiply an outbreeding variety without segregation of traits in the offspring, for example apples, pears, cherries and other fruits, potatoes, and bananas. Generally, these crops also have a long generation time so that breeding takes a decade or more. It also takes years for new varieties to reach a fair market share, after which this market share may remain constant for many years. This may make it attractive for breeders to “improve” the variety and extend its lifetime under Plant Breeders Rights and/or increase market share even further. When the variety is sold under a brand name, the “improved” variant can just replace the original one, as one does with varieties with selected random mutations.^128^

A possible additional deterrent in the developed of gene-edited plants are the high cost of the licenses on the CRISPR-technology. Developers need to acquire a license from the original applicants (CVC or the Broad) either directly or indirectly through larger companies that acquired several licenses.^128^ The number of licenses related to CRISPR technology and their high cost is a new development in the plant biotechnological field. Other licenses, such as those for TALENs and ZFNs have not encountered the same amount of dispute. The high cost of the license can potentially hinder adoption of the technology and commercialization of gene-edited products.

Gene editing technologies can add to the plant breeder’s toolbox in combating challenges that agriculture faces today, such as increasing disease pressure, the phasing out of chemicals, drought and increased temperatures. The success of these new crops, and the market share they will gain, will depend on various factors, that are similar to factors for conventionally bred crops, with the added stigma of being genetically modified.

## Conclusions

To conclude, only a few gene-edited products are currently on the market (predominantly focused on traits interesting to the end-consumer) or have been approved for commercial release. Many more products are in the pipeline or in research trials with various modified traits, some fitting in more sustainable crop systems, such as plants that require a lower pesticide use or are more climate-resilient. In the near future, we expect a gradual increase in gene-edited products on the market. It remains to be seen which of the products in the pipeline will have a successful market introduction and gain a market share. A successful introduction of a new variety not only depends on consumer acceptance, but also on other factors that conventional plant varieties and products also face, such as farmer and processor needs, competition with other varieties of the same crop and with similar, competing products, market dynamics, and distribution limitations.

## Methods

For the identification of commercialized or approved gene-edited plant products, and regulations of these products worldwide, the following sources were used: the website of the Genetic Literacy Project ^120^ and the website of ISAAA Inc.^129^, and four review (chapter-)books were consulted.^30,130–132^ Other review papers that have covered regulation worldwide extensively, have been consulted for this review.^133–135^

A systematic review of marketed and approved gene-edited plants and products worldwide is challenging due to unavailability of information for certain countries (notably South American countries and China). A number of countries have national databases for gene-edited plants and products, but is unclear whether they are always publicly accessible.

For GM products, the governmental websites of the USA ^136,137^ and Canada ^27,89^ were mainly consulted. For specific countries, governmental websites, USDA reports, or the above-mentioned review chapters were consulted.

The selected GM products, commercialized gene-edited products and gene-edited products in the pipeline were further confirmed by looking up news articles and pages of companies developing gene-edited products.

The GMO register of the European Commission was consulted for the GMO field trials in the EU (GMO register – Food Safety – European Commission).

## Data Availability

Data availability is not applicable. No new data have been generated.
